# Symmetry-Selective Ultrafast Charge Transfer via Cyano End Groups at the PDIF-CN_2_–Au(111) Interface

**DOI:** 10.1021/acs.nanolett.6c01061

**Published:** 2026-05-19

**Authors:** Gregor Kladnik, Antonio Cassinese, Luca Schio, Andrea Goldoni, Alberto Morgante, Luca Floreano, Dean Cvetko

**Affiliations:** † Faculty of Mathematics and Physics, 37663 University of Ljubljana, SI-1000 Ljubljana, Slovenia; ‡ Physics Department, University of Naples ‘Federico II’ and CNR SPIN, 80125 Naples, Italy; § CNR-IOM, Istituto Officina dei Materiali, Basovizza Area Science Park, 34149 Trieste, Italy; ∥ Sincrotrone Elettra, 34149 Trieste, Italy; ⊥ Physics Department, University of Trieste, 34127 Trieste, Italy; # Jožef Stefan Institute, SI-1000 Ljubljana, Slovenia

**Keywords:** organic−metal interfaces, cyano functionalization, ultrafast charge transfer, orbital symmetry selection, electronic transparency, interfacial electron dynamics, core-hole-clock spectroscopy, resonant Auger

## Abstract

Cyano-functionalized perylene diimides provide a platform for engineering orbital-specific electronic transparency at organic–metal interfaces. Here we resolve the adsorption geometry, orbital symmetry, and ultrafast charge-transfer dynamics of PDIF-CN_2_ on Au(111) using polarization-dependent NEXAFS, XPS, resonant Auger spectroscopy with core-hole-clock analysis, and STM. Molecules in the monolayer lie flat with out-of-molecular plane polarized cyano-derived π* orbitals aligned toward Au(111). Although the overall electronic structure remains largely molecular, the nearly degenerate cyano σ*­(π)/π*­(π) doublet becomes distinctly split at the interface, enabling symmetry-selective coupling of cyano orbitals. We use Auger spectator shifts to quantify bidirectional charge exchange within the N 1s core-hole lifetime, yielding injection of ∼0.43 e^–^ from Au into the cyano π*­(π) orbital and the transfer of ∼0.17 e^–^ in the opposite direction. These findings establish cyano end groups as symmetry-selective gateways for femtosecond charge transfer at molecule–metal interfaces.

## Graphical abstract

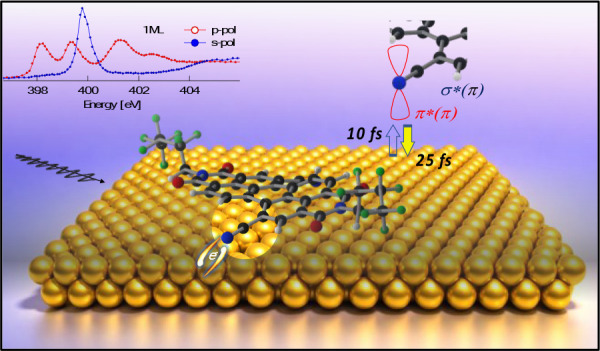

Molecular-scale control of charge transfer across organic–metal interfaces remains a central challenge in molecular electronics and hybrid quantum materials.^1^ Conjugated π-systems, functionalized with electron-withdrawing end groups, offer a powerful route to engineer interfacial electronic coupling while preserving intrinsic molecular properties.^2–4^ Among these, cyano-functionalized perylene diimides (PDIs) are particularly attractive due to their low-lying LUMO levels, environmental stability, and strong n-type character.^5,6^ PDIF-CN_2_, a high-mobility n-type perylene diimide derivative, combines a π-conjugated core with terminal cyano groups capable of mediating selective coupling to metal surfaces. While PDIF-CN_2_ and related compounds have been extensively investigated in thin-film electronics,^7–9^ a microscopic understanding of how cyano end groups control the orbital hybridization and charge-transfer dynamics at metal interfaces remains incomplete.^10–13^ In particular, neither the symmetry-selective orbital pathways nor the characteristic time scales of charge exchange have been directly resolved.

Previous NEXAFS studies have demonstrated that charge delocalization in donor–acceptor systems can be highly orbital-selective, with cyano-based π* orbitals playing a dominant role.^14–16^ Similarly, break-junction experiments and theoretical modeling on related PDI derivatives suggest that cyano groups can establish preferential high-conductance transport pathways by directly coupling to metallic electrodes.^17^ These findings highlight the importance of site- and symmetry-resolved approaches to disentangle interfacial charge-transfer mechanisms.

Here, we investigate the adsorption geometry, orbital hybridization, and ultrafast charge-transfer dynamics at the PDIF-CN_2_–Au­(111) interface. Using high-resolution X-ray photoelectron spectroscopy (XPS), polarization-dependent near-edge X-ray absorption fine structure (NEXAFS), resonant Auger electron spectroscopy (RAES) with core-hole-clock (CHC) analysis, and scanning tunneling microscopy (STM), we directly quantify the fractional charge transfer occurring within the nitrogen core-hole lifetime. We demonstrate that the cyano-derived π*­(π) orbital establishes a symmetry-selective, electronically transparent pathway for femtosecond charge exchange, whereas its orthogonal σ*­(π) orbital remains electronically opaque to interfacial charge transfer. These results reveal orbital-resolved bidirectional charge transfer at a weakly bonded organic–metal interface and establish the molecular end group symmetry as a governing principle for ultrafast interfacial coupling.

We examine the interfacial electronic structure and charge-transfer dynamics at the PDIF-CN_2_–Au­(111) interface using a multitechnique spectroscopic approach that combines high-resolution X-ray photoelectron spectroscopy (XPS), near-edge X-ray absorption fine structure (NEXAFS), and resonant photoemission spectroscopy (RPES).^18–20^ Thin PDIF-CN_2_ films were grown on atomically clean Au(111) surfaces by vacuum sublimation. The deposition rate was monitored in real time with a quartz crystal microbalance, and the actual film thickness was further verified *a posteriori* by the attenuation of the Au 4f core level signal in XPS.

Figure 1a shows the N 1s spectrum of a 2.5-layer thick (∼15.2 Å) PDIF-CN_2_ film on Au(111), which exhibits two main peaks assigned to imide nitrogen (*E*_imide_ = 400.5 eV) and cyano nitrogen (*E*_cyano_ = 399.0 eV). These values agree with reported binding energies for cyano nitrogen in benzonitrile^21^ and PDI8-CN_2_,^22^ as well as for imide nitrogen in PTCDI.^23^ Our findings are in good agreement also with the multilayer value of 401.1 eV, reported for imide linked to a pyridine group.^24^ Figure 1b presents the corresponding C 1s XPS spectrum, with well-resolved components corresponding to chemically distinct carbon environments: the perylene backbone, cyano (C≡N), imide (N–C=O), and fluorinated terminal groups (CF_2_ and CF_3_). These assignments are supported by spectral decomposition through fitting and are consistent with previously reported data for perylene diimide derivatives.^22,25^ The C 1s and N 1s XPS spectra of the monolayer reproduce the respective XPS spectra of the thick film with an overall binding energy shift of about 0.5 eV to lower values due to core-hole screening by the Au electronic band (see Figure S2).

**Figure 1:**
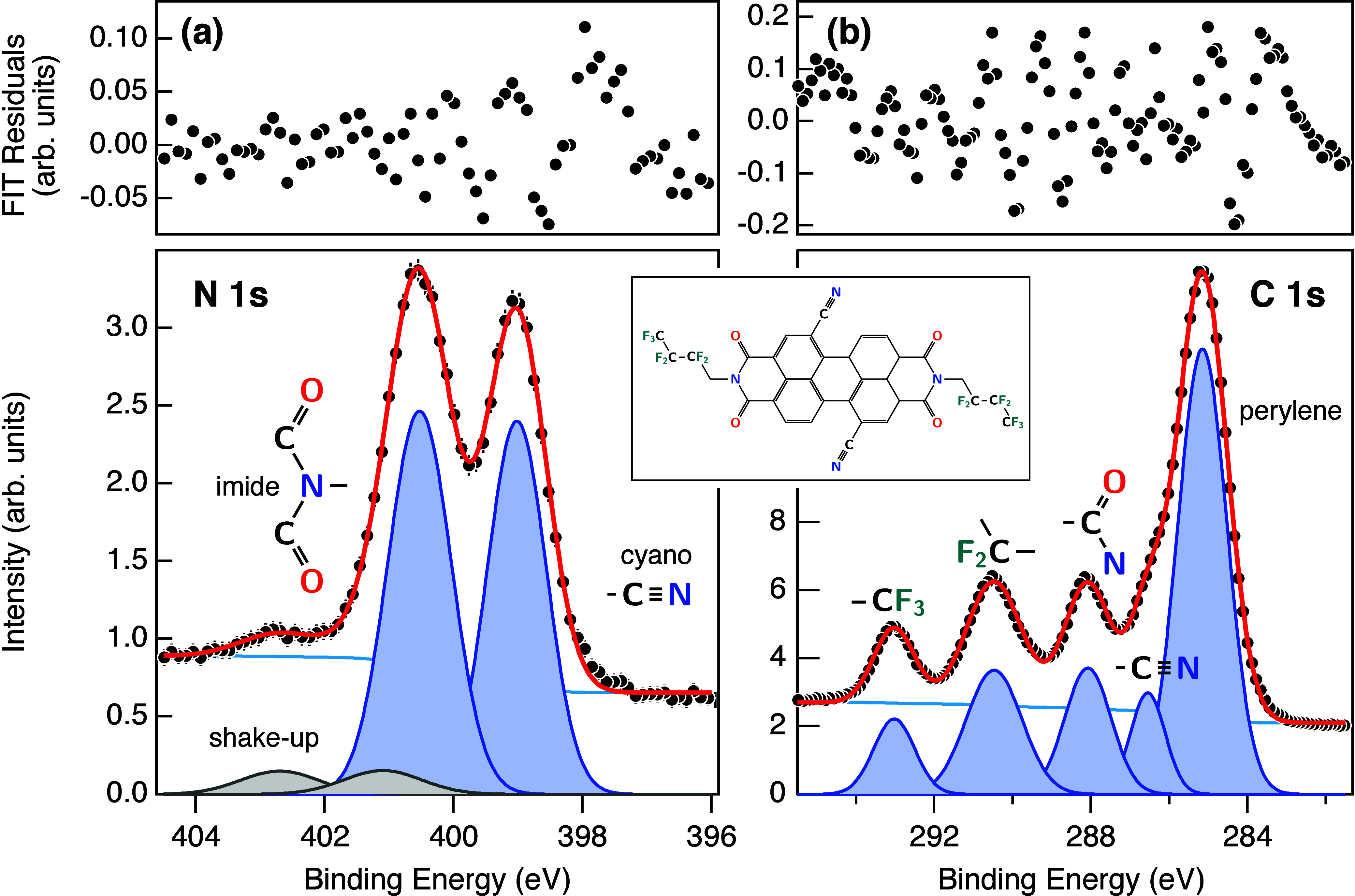
High-resolution X-ray photoelectron spectroscopy (XPS) data of 15.2 Å thick (∼2.5 ML) PDIF-CN_2_ on Au(111) taken with a photon energy of 500 eV. (a) N 1s XPS spectrum showing two main components corresponding to imide and nitrile nitrogen atoms. (b) C 1s XPS spectrum. Distinct components from chemically inequivalent carbon atoms are resolved: the perylene backbone, cyano (C≡N), imide (N–C=O), and fluorinated terminal groups (CF_2_ and CF_3_), as identified by spectral decomposition (molecular structure shown in the inset). For the details of the spectral decomposition and the fit of the monolayer film, see Figure S1 and Table S1.

We next employ STM imaging- and polarization-dependent NEXAFS to determine orbital coupling at the PDIF-CN_2_–Au­(111) interface. Figure 2a presents the nitrogen K-edge NEXAFS spectra of PDIF-CN_2_ films with nominal thicknesses of ∼0.9 monolayer (ML, ∼6.3 Å) and 2.5 ML on Au(111). Panels b and c of Figure 2 show the corresponding STM topographic images of the PDIF-CN_2_ contact layer, revealing molecules arranged in a nearly two-dimensional “herringbone”-like packing motif (see the Supporting Information for details). The molecular domains form a closely rectangular superlattice composed of two molecular pairs per unit cell, resulting in an overall molecular density of approximately 0.6 molecule nm^–2^.

**Figure 2:**
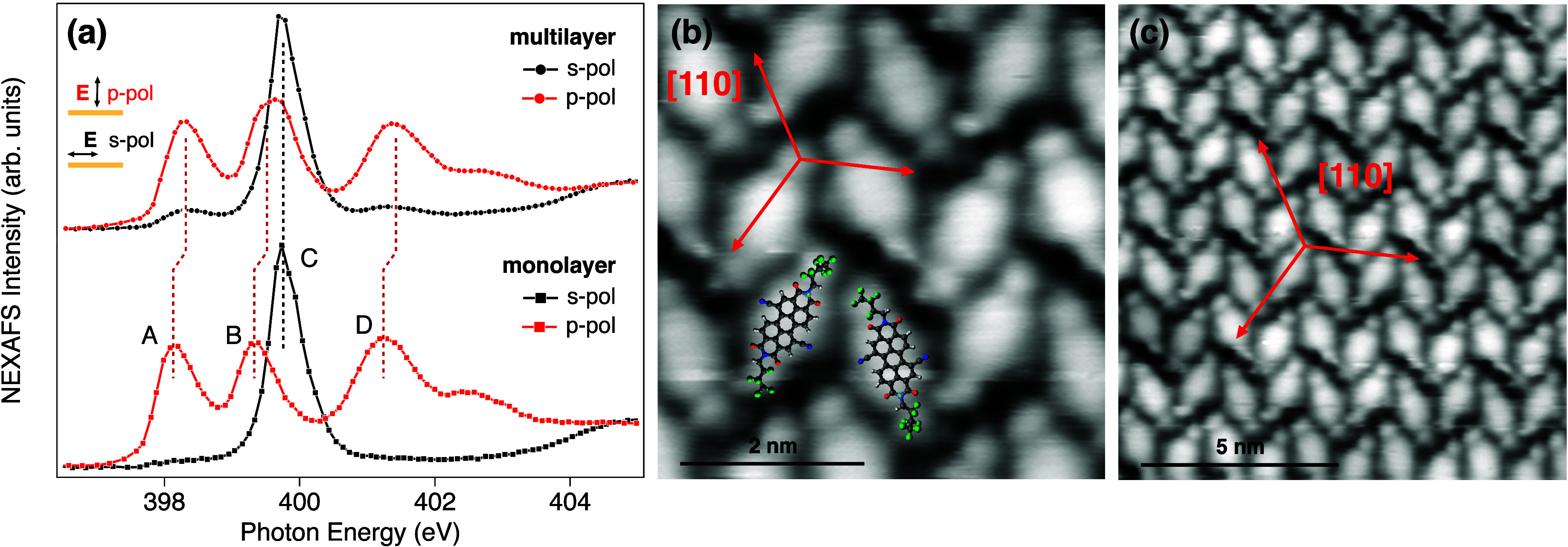
(a) Nitrogen K-edge NEXAFS spectra of PDIF-CN_2_ for a thick film (2.5 ML, top panel) and a contact monolayer (∼0.9 ML, bottom panel) on Au(111), measured with p-polarized (p-pol, red) and s-polarized (s-pol, black) light. The pronounced linear dichroism between p-pol and s-pol spectra demonstrates a flat-lying adsorption geometry of the molecules on Au(111). (b and c) STM topographic images taken at a bias voltage of −1.5 V and a tunneling current of 170 pA of the PDIF-CN_2_ contact monolayer showing flat-lying molecular adsorption with (b) orientational coupling and (c) long-range, ordered herringbone packing. See also Figures S7–S9.

The NEXAFS spectra were recorded using two complementary photon-polarization geometries: p-polarization (electric-field vector oriented along the surface normal, TM) and s-polarization (electric-field vector parallel to the surface, TE). We detect a pronounced polarization dependence of the NEXAFS resonance intensity in both the monolayer and 2.5 ML films, where the intensity of π*-symmetry resonances is highest in TM polarization and almost 1 order of magnitude lower in TE polarization (practically vanishing for the monolayer). Such a large NEXAFS linear dichroism indicates a preferential flat-lying molecular orientation with the aromatic system parallel to the surface, facilitating discrimination of the symmetry and localization of the molecular orbitals (see Figure 2 and Figure S4).

We first analyze the 2.5 ML thick film, whose limited thickness maintains the molecular orientation nearly parallel to the surface while exhibiting an electronic structure characteristic of multilayer PDIF-CN_2_. The lowest-energy resonances are associated with molecular orbitals localized on the cyano nitrogen atoms, whereas the imide nitrogen lies on the nodal plane of the PDI LUMO, independent of the terminal or peripheral functionalization of the PDI core.^26,27^ By analogy with tetracyanoquinodimethane (TCNQ), the first resonance (peak “A”) at ∼398.7 eV can be assigned to a π* orbital localized on the cyano nitrogen atom and extending over the adjacent aromatic carbon atoms.^28^ The next NEXAFS feature (peak “BC”) at ∼399.8 eV appears in both p- and s-polarization and corresponds to two π*-symmetry molecular orbitals associated with the C–N bond. These are orthogonal to the bond and to each other. In the isolated cyano molecule, they are degenerate, but upon coordination to the benzene ring, this degeneracy is lifted. One orbital lies in the plane of the aromatic ring, conventionally denoted σ*­(π), while the other is oriented out of plane, denoted π*­(π). Contributions from the imide nitrogen atoms emerge only at higher energy (401.7–402.8 eV, peak “D”), corresponding to π*-symmetry orbitals, overlapping with additional π* and σ* components from the cyano group and its adjacent aromatic ring.^26–28^

The PDIF-CN_2_ monolayer spectra exhibit the same overall NEXAFS resonance structure as the multilayer but with all π*-symmetry orbitals (both cyano- and imide-derived) shifted to lower energy by approximately 0.15 eV (see Figure S4 for details). This confirms that the PDIF-CN_2_ molecules in the monolayer maintain their molecular character as expected for monolayer films with no chemical coupling to the substrate. In contrast to more reactive metal substrates such as Ag and Cu, where the cyano LUMO resonance of TCNQ is completely quenched,^29–32^ the entire electronic structure of the cyano group is preserved on Au(111). Notably, the quasi-degenerate σ*­(π)/π*­(π) orbitals (peak “BC”) undergo substantial splitting due to their proximity to the surface, yet their relative ordering reverses compared with that typically observed for TCNQ on reactive substrates^29–32^ and even on Au(111) itself.^31^ In the monolayer, the π*­(π) orbital (peak “B”) shifts to lower energy while the σ*­(π) orbital (peak “C”) remains nearly the same as in the multilayer. This selective energy shift indicates that orbital hybridization with Au is favored when the orientation of the molecular orbital allows spatial extension toward the substrate. Such an interaction puts the CN π* orbital in an electronic configuration that promotes its participation, together with the aromatic backbone, in charge delocalization and transfer to the substrate.

Finally, the pronounced NEXAFS dichroism in the monolayer confirms that the PDIF-CN_2_ molecules adopt a perfectly flat-lying geometry, as the π- and σ-symmetry resonances vanish in s- and p-polarization, respectively. This orientation is optimal for maximizing electronic coupling between cyano-based molecular orbitals and the Au substrate. Such a geometry with π-symmetry orbitals provides the structural platform for orbital hybridization and electronic transparency at organometallic interfaces. In the following section, we directly probe this orbital-specific electronic transparency by measuring charge-transfer dynamics using resonant Auger photoemission within the core-hole-clock framework.

In core-hole-clock (CHC) analysis, the delocalization time of a photoexcited electron is commonly inferred from its competition with the core-hole decay lifetime: *τ*_ch_ = 5.4 ± 0.4 fs for N 1s.^33–35^ By analyzing the branching ratio of core-hole decay channels, we trace the localization dynamics of excited electrons across unoccupied molecular orbitals. We first consider the multilayer (2.5 ML) film, as representative of resonant spectroscopy on an effectively isolated molecules, serving as a reference system in which no charge transfer can occur.

A series of valence-band photoemission spectra were collected with the photon energy (*hν*) incrementally scanned across the N 1s absorption edge. For the sake of simplicity, we keep the notation of main NEXAFS resonances across the N K edge at *hν* values of ∼398.3, ∼399.7, and ∼401.4 eV as “A”, “BC”, and “D”, respectively. The resonant Auger electron spectroscopy (RAES) data set for the 2.5 ML thick PDIF-CN_2_ film is shown in Figure 3a as a two-dimensional false color intensity map *I*(*h*ν, *E*_k_), displaying photon (*hν*) and kinetic energy (*E*_k_) dependencies. The nonresonant background measured below the K edge (*hν* = 395 eV) was subtracted from each spectrum to highlight resonant contributions. We observe a broad resonant Auger feature emerging at *E*_k_ ∼ 377.7 eV, corresponding to the N 1s → LUMO resonance (*hν* ∼ 398.3 eV), with the intensity closely following the photon energy dependence of the NEXAFS signal across the 398–410 eV photon energy range. Above the ionization threshold (*hν* ∼ 404 eV) where the N 1s core electron is excited to the free electron continuum, the Auger line appears at a constant kinetic energy (^multi^ *E*_k_(free) ≈ 374.8 eV), corresponding to a final state comprising two valence-band holes (VB^2h^). Below the ionization potential (IP), the Auger lines recorded at the “A”, “BC”, and “D” resonances are Coulomb shifted to higher kinetic energies by *ΔE* ∼ 2.9 eV due to the Coulomb interaction with the localized “spectator” electron excited from the N 1s to unoccupied molecular orbitals (see the energy level diagram sketch in Figure 3). Here, this additional electron in the previously unoccupied orbital results in a final state comprising a single valence-band hole (VB^1h^). The magnitude of *ΔE* directly relates the spatial and temporal confinement of the core excited electron to the unfilled orbitals (LUMO, LUMO+1, etc.). It is largest for isolated molecules lacking orbital hybridization, whereas in strongly hybridized systems, efficient delocalization (transfer) of the excited electron occurs before the core-hole decay and the spectator shift disappears.

**Figure 3:**
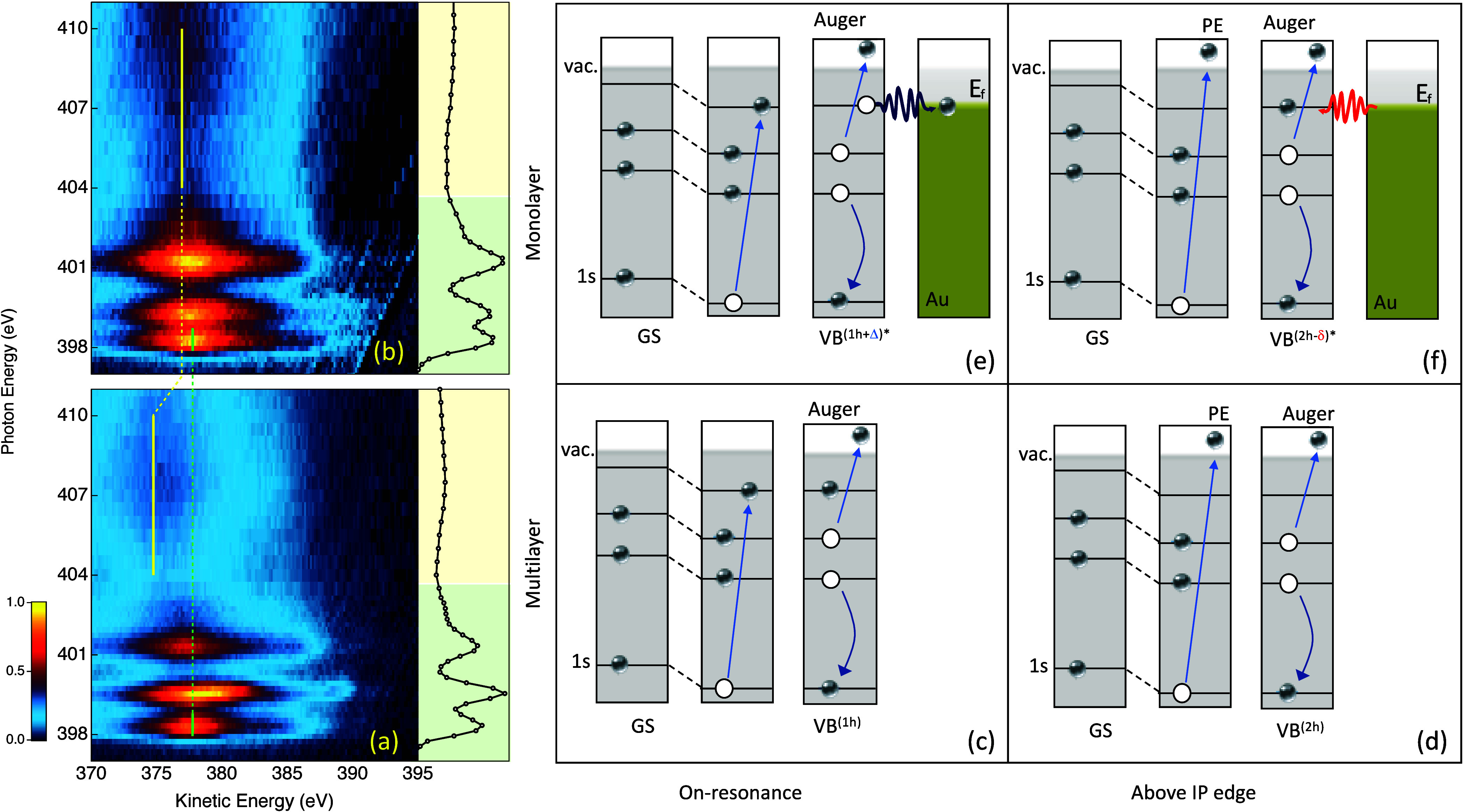
Nitrogen K-edge resonant Auger emission (RAES) maps displayed as a false color map function of photon energy (left axis) and electron kinetic energy (bottom axis) for (a) a 2.5 monolayer (ML) thick film and (b) an ∼0.9 ML contact layer of PDIF-CN_2_ on Au(111). Corresponding NEXAFS spectra (black lines) are shown on the right axes. The Auger peak positions at the LUMO resonance (*hν* = 377 eV) and above the ionization edge (*hν* > 404 eV) are indicated by green and yellow vertical lines, respectively. (c–f) Schematic energy level diagrams corresponding to excitations to LUMO and to the free continuum for multilayer and monolayer PDIF-CN_2_. Final states with the number of holes (h) in the valence band are labeled as 1h, 2h, 2h−δ, and 1h+Δ, where δ and Δ denote unitless fractions of electron charge normalized to elementary charge e_0_, transferred to and from the molecule, respectively.

The magnitude of *ΔE* found for our 2.5 layer thick PDIF-CN_2_ film indicates that cyano-based empty molecular orbitals are not hybridized, as expected for an isolated molecular system. Comparable spectator shifts of several electronvolts have been reported for uncoupled fullerenes (C_60_)^36^ and heterostacked molecular systems with amino–carboxylic coupling, where orbital-specific spectator shifts of ∼3.0 and ∼1.7 eV have been reported, corresponding to charge-transfer times of >50 and <20 fs, respectively.^37^ In this regard, it is worth noting that the Auger spectator corresponding to higher excitations at *hν* = 401.7 eV (resonance “D”) yields a slightly smaller energy shift (^multi^ *E*_k_(D) ≈ 2.5 eV). This smaller screening of the core hole localized on the imide atoms is expected because of the larger spatial delocalization of the orbital over the PDI heteroaromatic macrocycle with respect to that of the cyano­(-benzene) groups.

In stark contrast to the multilayer, the PDIF-CN_2_ monolayer (ML) on Au(111) exhibits markedly different charge-transfer behavior. At the “A”, “B”, and “D” resonances, where the core electron is excited into unoccupied molecular orbitals, the spectator Auger peak is centered at ^ML^ *E*_k_(A,B,D) ≈ 377.7 eV, which is similar to that observed for isolated molecules in the 2.5 ML film, reflecting a final state close to the single valence-band hole state (VB^1h^). Surprisingly, also when the core electron is photoexcited into the free-electron continuum above the ionization potential (*hν* = 404 eV), the Auger peak is nevertheless centered at an almost identical kinetic energy (^ML^ *E*_k_(free) ≈ 377 eV) so the final state is expected to be close to a state with a single hole in the valence band (VB^1h^). This implies that ultrafast charge injection from Au to the PDIF-CN_2_ molecule occurs on the time scale of N 1s core-hole decay.

To quantify the fraction of electron charge (δ) injected from Au into the molecule during the N 1s core-hole lifetime, we compare the kinetic energies (*E*_k_) of the Auger peaks in the monolayer and multilayer spectra, whereby we explicitly include also the effect of valence-band hole screening by the Au electronic states. The VB hole screening in the monolayer has been estimated separately from a comparison between valence-band photoemission spectra of PDIF-CN_2_ thick and monolayer films (see Figure S3).

Figure 4 compares the Auger spectra of PDIF-CN_2_ for thick and monolayer films on Au(111), measured at the nitrogen “A” resonance (*hν* = 398.3 eV) and at post-edge excitation into the free electron continuum (*hν* = 407 eV). For the thick film, the molecules behave as an electronically isolated moiety; thus, post-edge excitation yields a final state with two valence-band holes (VB^2h^). In contrast, for the monolayer, creation of the N 1s core hole transiently lowers the molecular LUMO toward the Au Fermi level, enabling bidirectional charge transfer between the Au electronic continuum and the PDIF-CN_2_ valence-band orbitals within the N 1s core-hole lifetime (see panels e and f of Figure 3).

**Figure 4:**
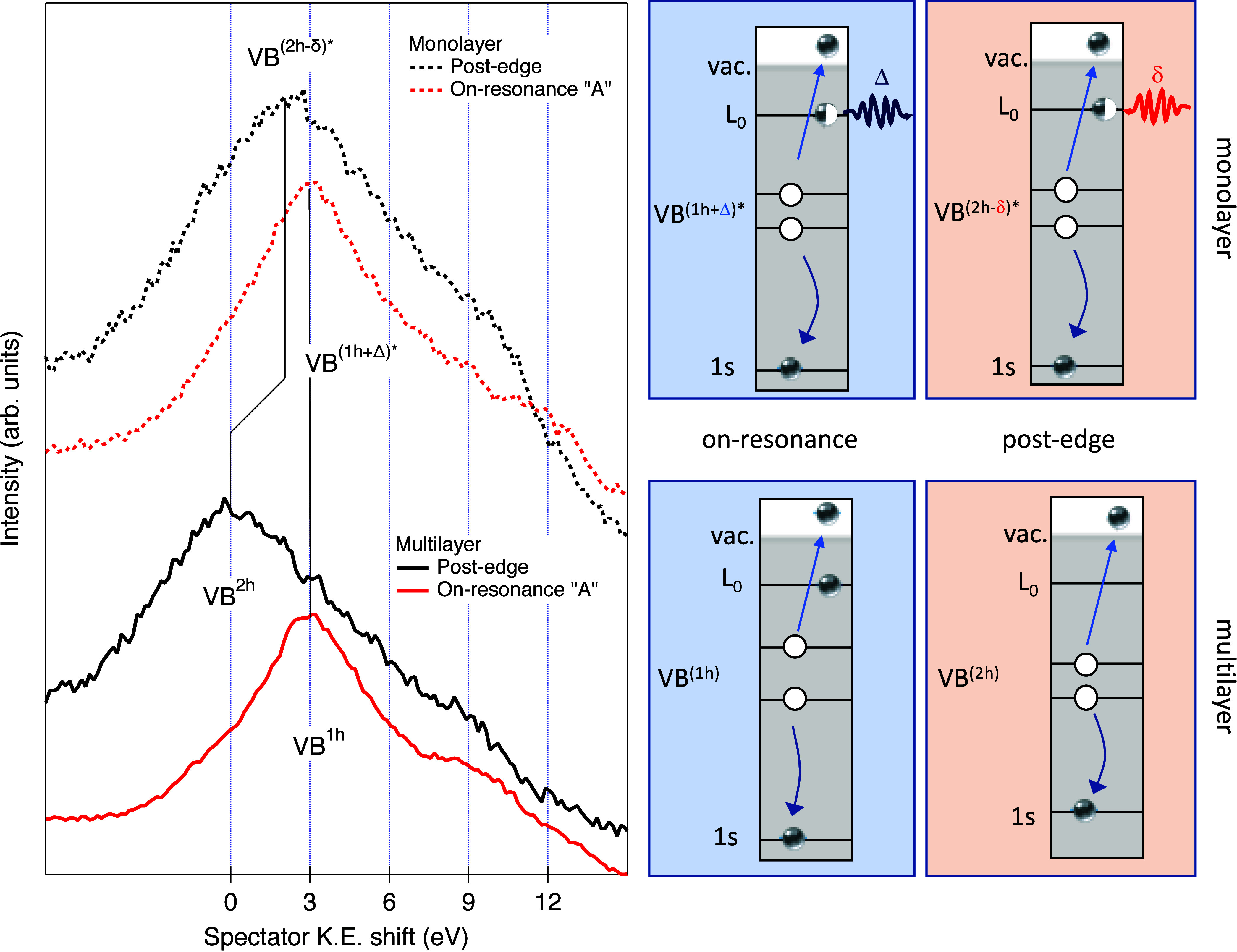
PDIF-CN_2_ Auger spectra taken at the N K edge for multilayer (∼2.5 ML, solid lines) and monolayer/Au(111) (∼0.9 ML, dashed lines). Spectra are measured at the nitrogen “A” resonance (*hν* = 398.3 eV (red)) and at post-edge excitation to the free continuum (*hν* = 407 eV (black)). The VB hole notation in the final state is indicated. Δ and δ refer to the unitless fractions of electron charge transferred from and to the PDIF-CN_2_ molecule in the monolayer, respectively.

The magnitude of the kinetic energy shift between the thick film and monolayer Auger peaks provides direct access to the amount of charge exchanged between PDIF-CN_2_ and Au(111) during the 5.4 ± 0.4 fs core-hole lifetime. From the kinetic energy difference between the on-resonance and post-edge Auger spectra in the multilayer (red and black solid lines, respectively, in Figure 4), we determine the Coulomb spectator shift associated with the presence or absence of one valence-band hole, since it also corresponds to the energy difference between the two final states of the molecule (*E*(VB^2h^) – *E*(VB^1h^) = 2.9 eV). We then compare the post-edge Auger spectra of the multilayer and the PDIF-CN_2_ monolayer (solid vs dashed black traces in Figure 4). The resulting shift of 2.2 eV measures final state difference *E*(VB^2h^) – *E*(VB^(2h‑δ)*^) and reflects the modified final state produced by fraction δ of electron charge transferred from Au to the molecule, together with screening (denoted with an asterisk) of the 2h-δ valence holes by the Au conduction electrons. By analyzing this spectator shift while explicitly accounting for the Au-induced screening (full derivation in eq 9 of the Supporting Information and Figure S5, as well as Figure S6 for the screening model), we obtain δ = 0.43 ± 0.06. Thus, on average, nearly half an electron is injected from Au into the cyano-localized π*­(π) orbital of PDIF-CN_2_ within the ∼5 fs N 1s core-hole lifetime.

A complementary analysis can be performed for the on-resonance Auger spectrum of the monolayer (red dashed line in Figure 4), where the N 1s electron is excited into the cyano-localized π*­(π) orbital. In this configuration, the excited electron may delocalize from the molecule into the Au electronic continuum prior to the core-hole decay (see Figure 3f). By comparing the kinetic energy of the on-resonance Auger peak in the monolayer with that of the multilayer and, using the same spectator shift analysis framework—including the Au screening of valence-band holes—we extract the fraction of electron charge (Δ) transferred in the opposite direction, from the cyano π* orbital of the molecule into the substrate (details in eq 14 of the Supporting Information). We obtain Δ = 0.17 ± 0.05, indicating that a significant, though smaller, fraction of the excited charge on the molecule delocalizes into the Au continuum prior to the core-hole decay. This result establishes that the PDIF-CN_2_/Au­(111) interface supports bidirectional, few-femtosecond charge exchange, selectively mediated by the cyano-anchored π* orbital. A full derivation of the relation between the measured kinetic energy shifts, valence-hole screening, and extracted fractions δ and Δ of electron charges transferred is provided in the Supporting Information.

Thus, within the N 1s core-hole lifetime of 5.4 ± 0.4 fs, we resolve two ultrafast cyano orbital-based channels transferring a fraction of electron charge in opposite directions: δ = 0.43 ± 0.06 for Au → π*­(π)_CN_, and Δ = 0.17 ± 0.05 for π*­(π)_CN_ → Au. Within the core-hole-clock (CHC) formalism, the charge-transfer time (*τ*_CT_) is expressed in units of the core-hole lifetime (*τ*_ch_) as $\tau_{\mathrm{CT}}=\tau_{\mathrm{ch}}\frac{P_{\mathrm{no CT}}}{P_{\mathrm{CT}}}$ , where *P*_CT_ and *P*_no CT_ denote the probabilities for charge transfer (CT) occurring and not occurring, respectively, prior to core-hole decay.^38^ Since CT probabilities are directly related to the transferred fraction of electron charge as $\frac{P_{\mathrm{no CT}}}{P_{\mathrm{CT}}}=\frac{1-\delta }{\delta }$ , the measured fractions of δ = 0.43 and Δ = 0.17 correspond to ultrafast electron injection from Au into the cyano π* orbital of PDIF-CN_2_ occurring in *τ*_CT_ (Au → PDIF-CN_2_) = 7.0_–1.5_^+2.2^ fs and to a slower electron delocalization from the molecule into the Au continuum of *τ*_CT_ (PDIF-CN_2_ → Au) = 27_–8_^+11^ fs (see the Supporting Information for details of the calculation and the uncertainty propagation method). A summary of the results is given in Table S2. The strength of the cyano π* orbital coupling to Au, which defines the degree of electronic transparency at the interface, may be compared with reported charge-transfer times in related hybrid systems. Ultrafast charge transfer has been measured for chemically bonded atomic sulfur on ruthenium (<0.3 fs),^39^ amine lone pair binding to Au (<1 fs),^40^ and intermolecular amino–carboxyl coupling (∼10 fs).^37^ Electron injection from the Fermi level of a metallic substrate into nitrogen-based molecular orbitals has also been reported for pyridine monolayers on Au(111), with charge-transfer times ranging from 3 to 30 fs, depending sensitively on the adsorption geometry and the orientation of the target molecular orbital relative to the Au(111) surface.^41^ Moreover, the critical role of molecular adsorption and conformation geometry in establishing efficient pathways for ultrafast electron injection has been demonstrated for direct electron transfer at hybrid organometallic interfaces.^42^

Ultrafast electron dynamics has been reported for through-space transfer in weakly coupled systems, e.g., shape-matched donor–acceptor complexes such as hexabenzocoronene (HBC)–C_60_ host–guest pairs (∼16 fs),^43^ as well as in encapsulated fullerenes,^44^ and in π–π stacked aromatic structures, such as paracyclophanes (PCPs), where the through-space charge-transfer time dramatically increases with the spatial separation between aromatic rings, ranging from ∼1–2 fs in [2,2]-PCP (2 Å spacing) to >50 fs in [4,4]-PCP (3 Å spacing).^45^ Similarly, sub-50 fs charge-transfer times have been demonstrated in weakly coupled WS_2_–MoS_2_ and MoSe_2_/WS_2_ van der Waals heterostructures, where electron–phonon coupling opens additional ultrafast charge-transfer pathways between layers despite otherwise weak electronic hybridization.^46–48^

In summary, polarization-dependent NEXAFS, STM, and resonant Auger spectroscopy show that the cyano-derived π*­(π) orbitals of PDIF-CN_2_ constitute the dominant electronic coupling channel at the PDIF-CN_2_–Au­(111) interface. The molecules adopt a flat-lying geometry that aligns π-symmetry orbitals toward the metal, enabling symmetry-selective interfacial hybridization. Interaction with Au lifts the near degeneracy of the cyano-derived σ*­(π)/π*­(π) doublet, which is unambiguously resolved using complementary NEXAFS polarization geometries.

Core-hole-clock analysis of resonant Auger spectra, based on spectator Auger energy shifts, provides a direct and novel route to quantify ultrafast, bidirectional charge exchange across the interface. Electron injection from Au into the π*­(π) orbital occurs on a sub-10 fs time scale, while charge transfer in the opposite direction proceeds on an ∼25 fs time scale. This establishes a symmetry-selective bidirectional charge-transfer pathway mediated exclusively by the π*­(π) component, whereas the σ*­(π) orbital remains electronically inactive with respect to interfacial charge transfer.

Overall, the cyano end groups of PDIF-CN_2_ act as an electronic gateway for ultrafast, symmetry-controlled charge transfer at the organometallic interface. The orbital-specific hybridization defines an electronically transparent junction in which molecular symmetry and orientation govern the femtosecond charge dynamics.This work establishes end group-engineered orbital selectivity as a design principle for controlling ultrafast charge transfer at weakly bonded molecular interfaces.

## Supplementary Material



## Notes

High-resolution N 1s and C 1s XPS spectra of monolayer PDIF-CN_2_ on Au(111) (Figure S1), N 1s and C 1s binding energy shifts between mono- and multilayer PDIF-CN_2_ films (Figure S2), comparison of the high-resolution valence-band photoemission of monolayer and multilayer PDIF-CN_2_ on Au(111) (Figure S3), comparison of magic angle nitrogen K-edge NEXAFS of monolayer and multilayer PDIF-CN_2_ (Figure S4), resonant Auger spectra taken at various photon energies corresponding to excitations to different LUMO resonances and to the free electron continuum with indicated peak positions (Figure S5), image charge screening potential model (Figure S6), STM topographic image of monolayer PDIF-CN_2_ on Au(111) (Figure S7), LEED image of the PDIF-CN_2_ monolayer on Au(111) and the LEED pattern of clean Au(111) (Figure S8), STM images of PDIF-CN_2_ films on Au(111) at increasing molecular coverages (Figure S9), obtained XPS fit peak positions and fwhm values for the monolayer and multilayer PDIF-CN_2_ films (Table S1), and summary of experimental values used for the calculation and the resulting fraction of electron charge transfer and charge-transfer times (Table S2) (PDF)
