# Supplementary material for: Symmetry-Selective Ultrafast Charge Transfer via Cyano End Groups at the PDIF-CN2–Au(111) Interface
Source: Nano Lett. 2026 May 19;26(21):6983–90. doi: 10.1021/acs.nanolett.6c01061 (PMC13237819; doi:10.1021/acs.nanolett.6c01061)
Supplement: Supplementary file 1 [file nl6c01061_si_001.pdf]

# Supporting Information

# Symmetry-Selective Ultrafast Charge Transfer via Cyano Endgroups at the PDIF-$CN_2$/Au(111) Interface

*Gregor Kladnik[1,3,*], Antonio Cassinese,[2,3] Luca Schio[3], Andrea Goldoni[4], Alberto Morgante[3,5], Luca Floreano[3,*], and Dean Cvetko[1,3,6,*]*

[1] Faculty of Mathematics and Physics, University of Ljubljana, SI-1000 Ljubljana, Slovenia.

[2] Physics Department, University of Naples 'Federico II' and CNR SPIN, 80125 Naples, Italy

[3] CNR-IOM, Istituto Officina dei Materiali, Basovizza Area Science Park, 34149 Trieste, Italy.

[4] Sincrotrone Elettra, 34149 Trieste, Italy

[5]Physics department, University of Trieste, 34127 Trieste, Italy.

[6]Jožef Stefan Institute, SI-1000 Ljubljana, Slovenia.

## Corresponding Authors

* Luca.Floreano@iom.cnr.it, dean.cvetko@fmf.uni-lj.si, gregor.kladnik@fmf.uni-lj.si

# Methods

PDIF-$CN_2$ powders (commercially known as ActivInk N1100) were purchased from Polyera Corporation (now Flexterra).

## Scanning tunnelling microscopy

The scanning tunnelling microscopy (STM) measurements were performed with Aarhus type Specs microscope (model 150) at the CNR-IOM/Elettra joint laboratory for microscopy (OSMOS) in Trieste, Italy. The microscope is attached to an UHV preparation chamber equipped with evaporation cells and an $Ar^+$ ion sputter gun. The sample holding manipulator arm hosts a heating unit for thermal annealing with temperature control and a quartz microbalance for evaporation rate monitoring.

Prior to each deposition of molecules, the Au surface was cleaned by at least two cycles of $Ar^+$ ion sputtering (1.5 keV) followed by thermal annealing to 720 K to obtain a clean and well-ordered (111) surface with sufficiently large terraces (~100 nm) that exhibit a distinct herringbone reconstruction. After annealing, the surface was allowed to cool down and kept at RT during each deposition. The PDIF-$CN_2$ molecules were vacuum deposited from laboratory made evaporating cells, each consisting of a boron nitride ceramic crucible with a tantalum filament wrapped around it and a K-type thermocouple for temperature control. To remove the impurities, the evaporation cells containing the PDIF-$CN_2$ were degassed for several hours before the first deposition. The typical PDIF-$CN_2$ evaporation temperature was 510 K with typical rate between 0.1 and 0.3 Å/min. The sample was transferred to the STM device and analyzed immediately after each deposition.

All topographic images were acquired with the STM operating at RT and a chamber pressure in the range of $10^{-10}$ mbar. We used the STM in constant current mode ($I = 100$–$800$ pA) with a

tungsten tip and negative sample bias. The lateral scale calibration of the images was performed a posteriori by taking the Au(111) surface lattice constants as a reference.

## XPS, NEXAFS, and RAES spectroscopy

The XPS, NEXAFS, and RPES measurements were performed at the ALOISA beamline of the Elettra Synchrotron Facility, Trieste.[1] The Au(111) substrate surface was cleaned by repeated cycles of $Ar^+$ sputtering (1.5 kV) and annealing up to 720 K. The PDIF-$CN_2$ molecules were evaporated from a boron nitride crucible at typical temperatures of 515-520 K, corresponding to a deposition rate of approximately 30 min/monolayer at the sample position (crucible-sample distance 0.27 m). The total pressure during deposition (with the sample at room temperature) was 2-3 × $10^{-9}$ mbar. The quartz crystal microbalance (QCM) was operated at room temperature with a nominal molecular density of 1.42 Å/$cm^3$, which corresponds to a complete wetting monolayer at an estimated thickness of 6.5 – 7.0 Å. This value was determined a posteriori by XPS-based film thickness analysis on Au(111), as well as by multilayer thermal desorption measurements on Ag(111). The same sample preparation protocol was initially used in the STM setup, which is equipped with an identical boron nitride crucible, QCM, and comparable crucible-sample distance. The chemical composition of the PDIF-$CN_2$ layers was regularly checked during and after vacuum deposition by quantitative XPS.

The overall film thickness of the deposited molecules have been determined from the intensity attenuation of the Au 4f peak due to inelastic scattering of photoelectrons passing through the organic overlayer.[2] Core level photoemission data were acquired in normal emission geometry with a constant 4° grazing angle of linearly p-polarized light beam with respect to the Au(111) surface plane. The C 1s and N 1s XPS spectra were acquired at a photon energy of 500 eV and

pass energy of 15 eV with a total energy resolution of 160 meV. Binding energies were calibrated with respect to the bulk spectral component of the Au $4f_{7/2}$ peak at (84.02 ± 0.01) eV.[3] C K-edge and N K-edge NEXAFS spectra were acquired in partial electron yield mode by means of a channeltron multiplier equipped with a negatively biased grid (-250 V and -370 V, respectively) to filter out low energy secondary electrons, in order to improve the signal to background ratio. NEXAFS spectra were acquired at two different orientations of the surface plane with respect to linearly polarized synchrotron beam to probe the sample linear dichroism, namely transverse magnetic (TM, close to p-polarization) and transverse electric (TE, s-polarization) geometry, by sample rotation around the photon beam axis. The beam grazing angle was kept constant at 6°. The resolving power of the beamline at the N 1s absorption threshold has been reported to reach values up to 10000, while under standard operating conditions it is typically around 5000.[1] This corresponds to an overall energy resolution at the nitrogen K-edge of approximately 80 meV. The precision of the energy calibration, however, is primarily related to the reproducibility of the monochromator, which is on the order of 2 meV at 400 eV photon energy. The photon energy calibration and photon flux normalization methods are described in detail elsewhere.[4] Resonant Auger photoemission (RAES) was performed by acquisition of a series of photoemission spectra across the nitrogen K-edge with the photon energy tuned in steps of 0.1 eV through the N 1s absorption edge. The sample was kept at a grazing angle of 4° in TM geometry (nearly p-polarization), with the electron analyzer oriented in near normal emission. At each photon energy $h\nu$, single photoemission spectra (XPS) were measured in a ~60 eV binding energy window from the Fermi energy. The RAES measurements were performed by translating the sample transverse to the photon beam after each individual XPS scan in order to minimize any radiation damage, which is enhanced near the ionization threshold. All spectra have been merged in false color RPES

intensity maps represented in kinetic energy (KE) scale in the form of $I(h\nu, \text{KE})$. To highlight the resonant contribution in the photoemission spectra, the non-resonant component (due to direct photoemission from molecular orbitals and from the substrate) has been measured at $h\nu = 395$ eV for the N 1s RPES (below the resonant edge) and subtracted from all spectra across the edge. All measurements for the monolayer were carried out with the sample temperatures close to 300 K and at about 180 K for the multilayer. At room temperature the PDIF-$CN_2$ growth proceeds with Stranski-Krastanov growth mode with 3D island formation beyond the first contact monolayer. At 180 K the interlayer diffusion is suppressed, resulting in a uniform multilayer thickness of the PDIF-$CN_2$ films.

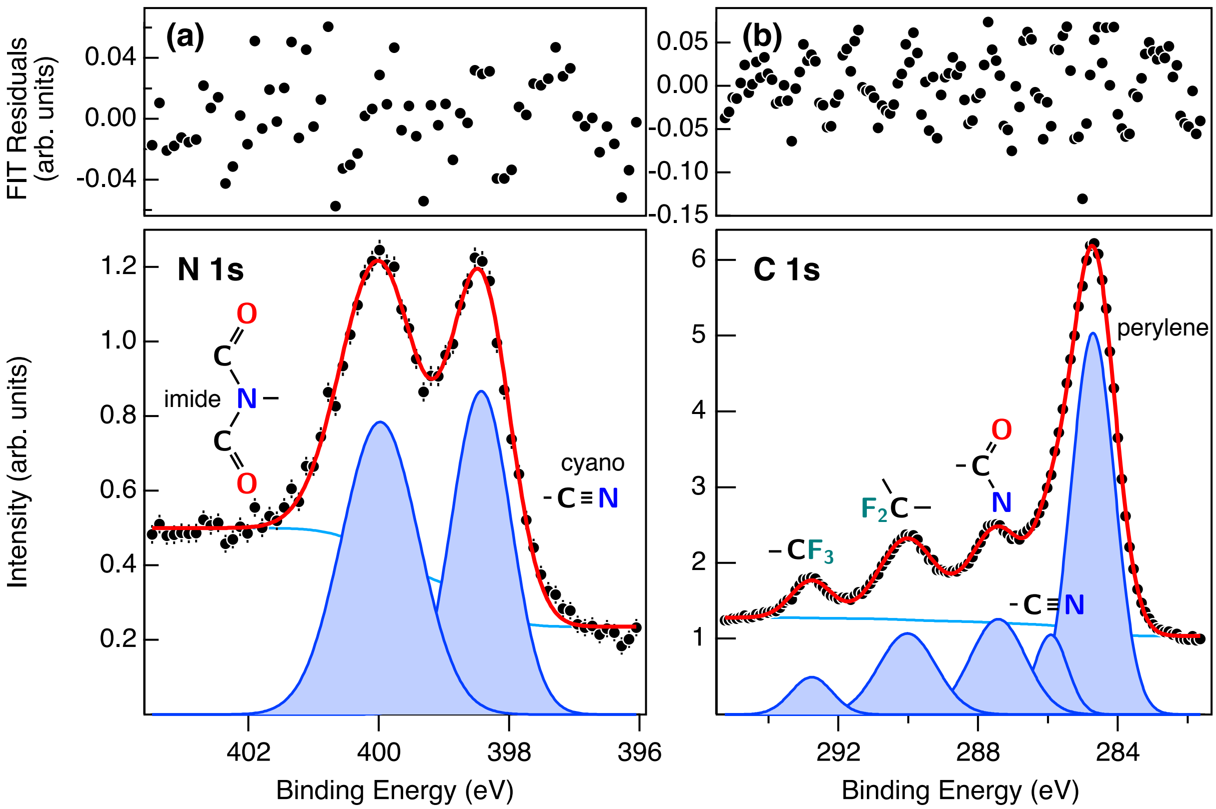


**Figure S1.** Fits of the N 1s and C 1s high-resolution X-ray photoelectron spectroscopy (XPS) spectra of monolayer (close to full monolayer, ~0.9 ML) PDIF-$CN_2$ on Au(111) taken at hν = 500 eV. Similar to the fits of the multilayer (Figure 1 in main paper) we resolve all the main components, but with a distinct binding energy shift to lower energies, see also Figure S2 for comparison. For the fits Gaussian peak shapes with an integrated (Shirley) background were used together with a constant offset. The amplitude of the integrated (Shirley) background was assumed to be the same for all the peaks in each spectrum.

**Table S1.** Obtained fit peak positions and FWHM values for the multilayer and monolayer N 1s and C 1s high-resolution X-ray photoelectron spectroscpy (XPS) spectra.

| peak | multilayer position (eV) | multilayer fwhm (eV) | monolayer position (eV) | monolayer fwhm (eV) |
|---|---|---|---|---|
| cyano N | $399.02 \pm 0.02$ | $1.08 \pm 0.01$ | $398.43 \pm 0.02$ | $1.07 \pm 0.03$ |
| imidic N | $400.53 \pm 0.02$ | $1.16 \pm 0.02$ | $399.98 \pm 0.03$ | $1.39 \pm 0.04$ |
| satellite | $402.66 \pm 0.11$ | $1.45 \pm 0.18$ | | |
| satellite | $401.06 \pm 0.12$ | $1.45 \pm 0.17$ | | |
| perylene | $285.15 \pm 0.02$ | $1.47 \pm 0.01$ | $284.71 \pm 0.02$ | $1.47 \pm 0.01$ |
| cyano C | $286.55 \pm 0.02$ | $1.05 \pm 0.03$ | $285.92 \pm 0.04$ | $1.13 \pm 0.02$ |
| imidic C | $288.08 \pm 0.02$ | $1.37 \pm 0.02$ | $287.43 \pm 0.03$ | $1.87 \pm 0.06$ |
| $CF_2$ | $290.48 \pm 0.02$ | $1.65 \pm 0.01$ | $290.03 \pm 0.03$ | $1.93 \pm 0.04$ |
| $CF_3$ | $293.02 \pm 0.02$ | $1.17 \pm 0.01$ | $292.77 \pm 0.03$ | $1.38 \pm 0.05$ |

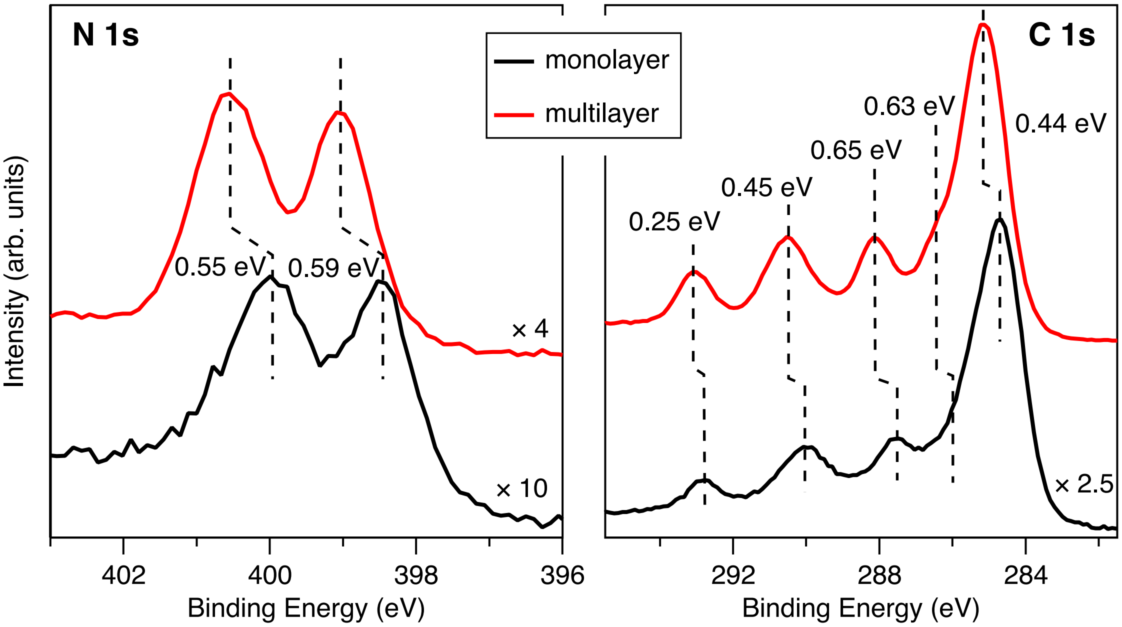


**Figure S2.** N 1s and C 1s binding energy shifts between mono- and multilayer PDIF-$CN_2$ film. Comparison of N 1s and C 1s spectra from a thick film (~2.5 ML, red) and a monolayer (~0.9 ML, black), measured with 500 eV photon energy. Vertical dashed lines indicate the observed energy shifts as deduced from the spectral fits.

Figure S2 compares the N 1s and C 1s XPS spectra of a contact monolayer (close to full monolayer, ~0.9 ML) with those of the thicker film (~2.5 ML). We observe a negative binding energy shift of approximately -0.5 to -0.6 eV (N 1s) and -0.4 to -0.6 eV (C 1s) in the contact monolayer relative to the thicker film. We attribute these shifts to final-state screening of the photoemission core hole by conduction electrons in gold.

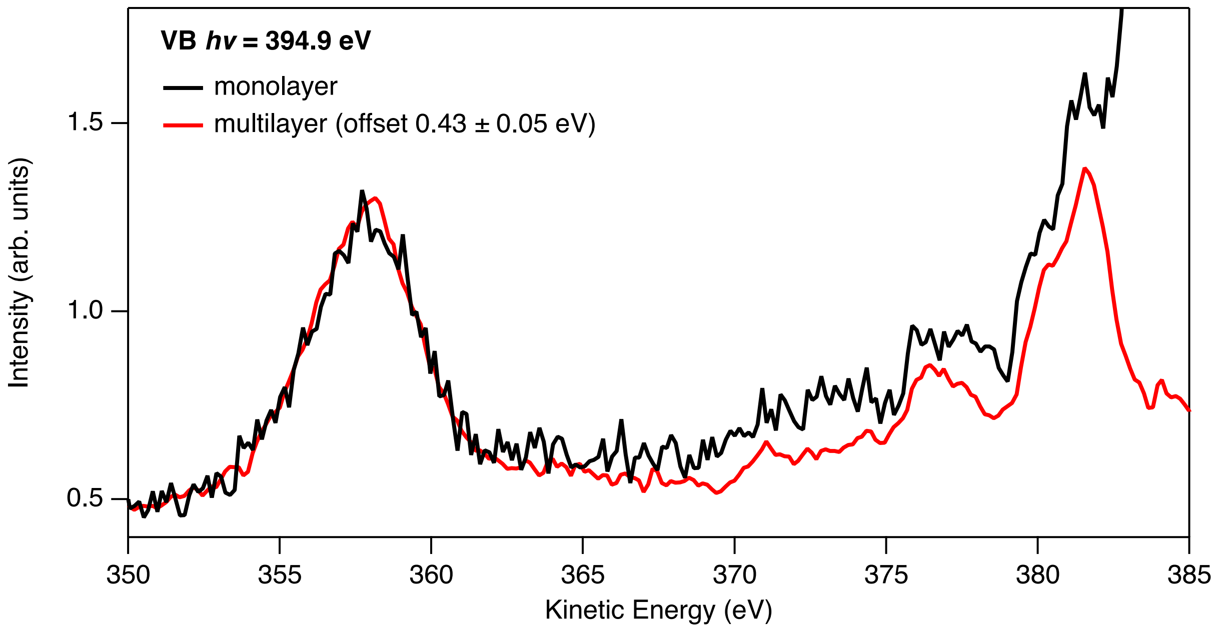


**Figure S3.** Comparison of high-resolution valence band photoemission with $h\nu$ = 394.9 eV (off-resonance, below N *K*-edge) for contact monolayer (close to full monolayer, ~0.9 ML, black) with that of the thicker film (~2.5 ML, red). A kinetic energy shift of (0.43 ± 0.05) eV is observed for VB peaks in the contact layer, attributed to the final screening of the VB hole by the Au conduction electrons.

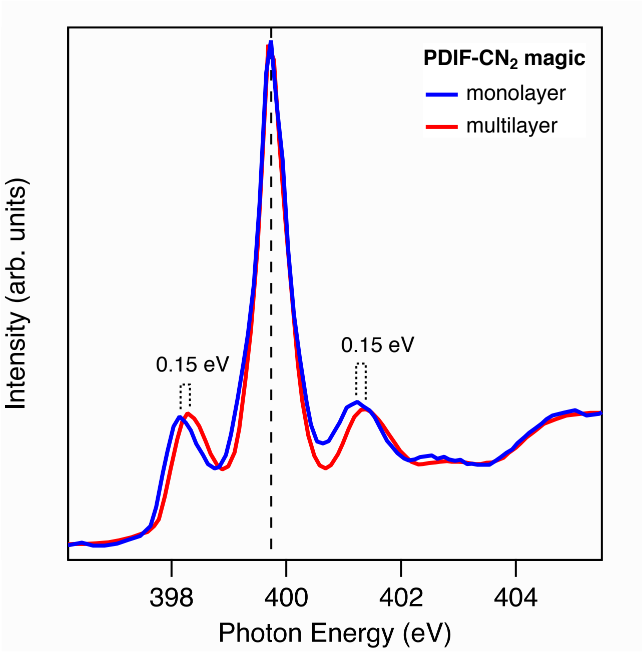


**Figure S4.** Magic angle Nitrogen K-edge NEXAFS spectra for PDIF-$CN_2$ monolayer (~0.9 ML, blue) and multilayer (~2.5 ML, red) on Au(111). A distinct energy shift of (0.15 ± 0.02) eV is observed for the π* transitions in the monolayer, but not for the σ*, indicating selective coupling of cyano-based π* orbitals with the Au(111) electronic band.

Figure S4 compares magic angle N K-edge NEXAFS spectra of monolayer (blue) and multilayer (red) PDIF-$CN_2$. We observe an energy shift of (0.15 ± 0.02) eV for the π* transitions in the monolayer. Relative energy calibration of both spectra has an uncertainty better than 2 meV.

# Auger spectator shift analysis

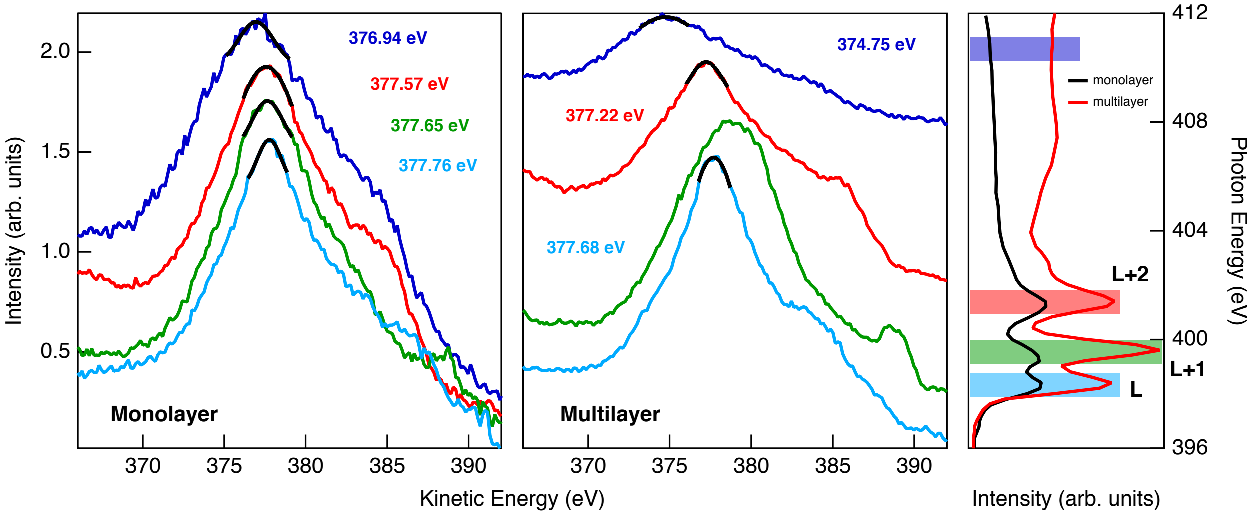


**Figure S5.** Resonant Auger spectra (RAES) at N 1s -> LUMO (light blue), LUMO+1 (green), LUMO+2 (light red) and to free electron continuum excitations (dark blue) for PDIF-$CN_2$ monolayer (~0.9 ML) and multilayer (~2.5 ML). The fitted Auger peak maxima are indicated (black) with the values obtained from the fit (all values ± 0.05 eV). We used a Gaussian peak shape to fit the Auger peak maxima in a narrow (~2 eV) range around the peak position. To the right the corresponding N K-edge NEXAFS is presented with indicated regions where the RAES spectra were taken.

# Image charge screening potential model

Evanescent electrons are subjected to additional electric field due to image charges in the metallic substrate. A negative image charge ($-ne_0$), fixed in space at $-z_0$, screens the molecular charge $ne_0$ (final charge state), effectively increasing the kinetic energy of the evanescent electrons, yet another (positive) image charge ($+e_0$) at $-z$ screens the evanscent electron itself, which is thus affected by an additional attractive force decreasing its kinetic energy, Figure S6.

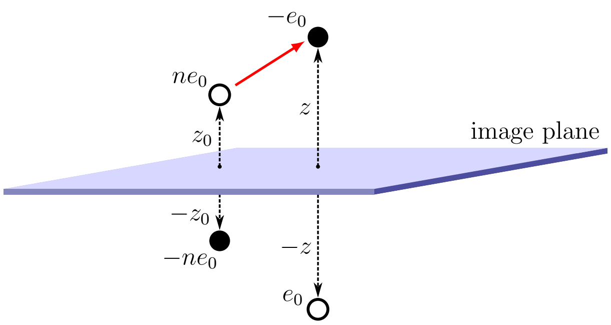


**Figure S6.** The in-space fixed molecular charge (final charge state $ne_0$) at $z_0$ and the evanescent electron at $z$ above the image plane are screened by their image charges.

The overall screening can be calculated in terms of classical electrostatic theory from the electric field at the current position $z$ of the evanescent electron, with elementary charge $e_0$, molecular charge (final charge state) $ne_0$, and dielectric constant $\varepsilon_0$, as

$$E(z) = \frac{e_0}{4\pi\varepsilon_0}\left(\frac{1}{(2z)^2} - \frac{n}{(z+z_0)^2}\right) \tag{1}$$

where the first term describes the electric field of the evanescent electron image charge and the second term the electric field of the molecular image charge. The total screening potential $\varphi$ equals to the path integral of the electric field from the initial evanescent electron position $z_0$ to infinity

$$\varphi = \int_{z_0}^{\infty} E(z)dz = \Delta E_{screen}(n) = -\frac{e_0}{4\pi\varepsilon_0}\frac{2n-1}{4z_0} \tag{2}$$

For example, a final charge state of *1h* ($n = 1$), e.g. VB/core-level photoemission, yields a screening potential of

$$\Delta E_{screen}(1h) = -\frac{e_0}{4\pi\varepsilon_0}\frac{1}{z_0} \tag{3}$$

whereas for a final charge state of *2h* ($n = 2$), e.g. normal Auger decay, we get a screening potential of

$$\Delta E_{screen}(2h) = -\frac{e_0}{4\pi\varepsilon_0}\frac{3}{4z_0} = 3\Delta E_{screen}(1h) \tag{4}$$

which is three-times larger than for a single hole ($1h$).

## Electron injection from Au(111) to PDIF-$CN_2$

To quantify the unitless fraction of electron charge $\delta$ (defined as amount of charge normalized to the elementary charge) injected from Au into the molecule during the N 1s core hole lifetime, we compare the kinetic energies ($E_k$) of the Auger peaks in the monolayer (ML) and multilayer (multi) spectra, whereby we explicitly include also the effect of valence-band hole screening by the Au electronic states. The VB hole screening in the molecular VB has been estimated separately from the energy shift of the valence-band photoemission spectra between the thick and monolayer films (see Figure S3).

Figure 4 in the main paper compares the Auger spectra of PDIF-$CN_2$ for both thick and monolayer films on Au(111), measured at the nitrogen "A" resonance (lowest unoccupied molecular orbital - LUMO, $h\nu$ = 398.3 eV) and at post-edge excitation into the free continuum ($h\nu$ = 407 eV). The difference in measured kinetic energy of the Auger peaks corresponds to the total energy difference of the respective final states. For the thick film, the molecules can be considered electronically isolated, and post-edge excitation leaves the system in a valence-band state containing two holes ($VB^{2h}$). In the monolayer film, the creation of a core hole dynamically lowers the $^{ML}$LUMO near the Au Fermi level, enabling (bidirectional) charge transfer between the Au substrate and the molecular valence-band orbitals within the core-hole decay timescale (See Figure 3e-f). In particular, we assume that upon post-edge excitation (IP) to the free electron continuum, a fraction $\delta$ of electron charge is transferred from Au $\rightarrow$ molecule. This charge transfer, together with

valence-band hole screening by the Au (charge state marked with asterisk - *), leads to the observed kinetic-energy shift between the respective Auger peaks, expressed as

$$E\left(VB^{(2h-\delta)^*}\right) - E(VB^{2h}) = {}^{ML}E_k(free) - {}^{multi}E_k(free) = 2.2\ eV \tag{5}$$

In a first approximation, we may neglect the cross-correlation effects between multiple holes in the VB states, hence assuming that the Auger shift follows a classical electrostatic model scaling with the effective charge of the VB states (*2h-δ*). When considering the additional screening of the VB holes by the Au surface electrons, we may further neglect that different spatial spread of VB molecular orbitals in the presence of a core hole may lead to slightly different screening of different VB holes, because the spatial spread of molecular VB orbitals in the presence of a core hole remains strongly localized on the specific excited atom. The screening of single valence-band holes in monolayer PDIF-$CN_2$ by Au(111) has been determined from valence-band photoemission as $\Delta E_{screen}(1h)$ = (0.43 ± 0.05) eV (see Figure S2).

From Eq. (2) with $n = 2\text{-}\delta$, the overall screening potential of the (*2h-δ*) final charge state leads to a shift in kinetic energy of emitted Auger electrons

$$E\left(VB^{(2h-\delta)^*}\right) - E\left(VB^{(2h-\delta)}\right) = \Delta E_{screen}(2h - \delta) = (3 - 2\delta) \cdot \Delta E_{screen}(1h) \tag{6}$$

where $E(VB^{(2h\text{-}\delta)})$ and $E(VB^{(2h\text{-}\delta)*})$ denote final state energies of PDIF-$CN_2$ with 2h-δ holes in the VB for an isolated layer and an adsorbed monolayer screened by Au(111), respectively.

Combining Equations (5) and (6) gives

$$E\left(VB^{(2h-\delta)}\right) - E(VB^{2h}) = {}^{ML}E_k(free) - {}^{multi}E_k(free) - (3 - 2\delta) \cdot \Delta E_{screen}(1h) \tag{7}$$

For $\delta = 1$, the left-hand side of Eq. (7) equals the bare Coulomb shift of the Auger peak induced by the spectator electron (e.g. on-resonance excitation in the thick film). For $\delta = 0$, the difference ${}^{ML}E_k(free) - {}^{multi}E_k(free)$ corresponds to the shift induced by the Au screening of two valence holes (see Eq. (4)). By proportionality, the spectator shift scales with the fraction $\delta$ of electron charge

transferred. We may also correlate the left term of Eq. (7) with the energy shift of the spectator Auger with respect to the Auger above IP as exclusively due to the fraction $\delta$ of electron charge transferred, without Au screening, as follows:

$$E\left(VB^{(2h-\delta)}\right) - E(VB^{2h}) = \left[{}^{multi}E_k(LUMO) - {}^{multi}E_k(free)\right] \cdot \delta \tag{8}$$

and therefore from Eqs. (7) and (8) we get

$$\delta = \frac{{}^{ML}E_k(free) - {}^{multi}E_k(free) - 3\Delta E_{screen}(1h)}{{}^{multi}E_k(LUMO) - {}^{multi}E_k(free) - 2\Delta E_{screen}(1h)} \tag{9}$$

Inserting the experimental values of the Auger peak positions from Figure 4 into Eq. (9) yields $\delta = 0.43 \pm 0.06$, indicating that within the nitrogen core-hole lifetime approximately 0.43 electron is transferred from Au to the cyano-based π* orbital of PDIF-$CN_2$.

## Electron transfer from PDIF-$CN_2$ to Au(111)

In a similar fashion to the analysis of electron injection from Au(111) to PDIF-$CN_2$, we analyse the Auger peak positions on the first resonance (peak "A", LUMO) in the multi and monolayer to obtain the fraction of electron charge $\Delta$ (defined as amount of charge normalized to the elementary charge) transferred *from* the molecule *to* the gold substrate.

First we note, that the total energy difference of the screened (*) final state with fraction $\Delta$ of electron charge transferred from the molecule to the substrate (i.e. adsorbed monolayer) and the unscreened final state with no charge transfer (multilayer) is given by the kinetic energy difference of the Auger peaks on the LUMO (peak "A") resonance as

$$E\left(VB^{(1h+\Delta)^*}\right) - E(VB^{1h}) = {}^{ML}E_k(LUMO) - {}^{multi}E_k(LUMO) \tag{10}$$

where we write the final charge state ($2h-(1\text{-}\Delta)$) equivalently as ($1h+\Delta$) for clarity.

Further we assume, as before, that the electron (hole) screening by the substrate is proportional to the charge on the molecule; thus from Eq. (2) with $n = 1+\Delta$, we may rewrite Eq. (6) as

$$E\left(VB^{(1h+\Delta)^*}\right) - E\left(VB^{(1h+\Delta)}\right) = \Delta E_{screen}(1h+\Delta) = (1+2\Delta)\cdot\Delta E_{screen}(1h) \tag{11}$$

Combining Eqs. (10) and (11) gives the total energy difference of two unscreened final states, one with no fractional charge transfer and one with fractional charge transfer as

$$E\left(VB^{(1h+\Delta)}\right) - E\left(VB^{1h}\right) = {}^{ML}E_k(LUMO) - {}^{multi}E_k(LUMO) - (1+2\Delta)\cdot\Delta E_{screen}(1h) \tag{12}$$

The same total energy difference can be expressed similarly to Eq. (8) as

$$E\left(VB^{(1h+\Delta)}\right) - E\left(VB^{1h}\right) = \left[{}^{multi}E_k(free) - {}^{multi}E_k(LUMO)\right]\cdot\Delta \tag{13}$$

Finally, combining Eqs. (12) and (13) and solving for $\Delta$ yields

$$\Delta = \frac{{}^{ML}E_k(LUMO) - {}^{multi}E_k(LUMO) - \Delta E_{screen}(1h)}{{}^{multi}E_k(free) - {}^{multi}E_k(LUMO) + 2\Delta E_{screen}(1h)} \tag{14}$$

Inserting the measured kinetic energies of the Auger peaks and the valence band screening shift into Eq. (14) we obtain the fraction of electron charge transferred from the molecule to the Au substrate of $\Delta = 0.17 \pm 0.05$. A summary of the measured Auger kinetic energies and the obtained charge transfers are given in Table S2.

## Uncertainty estimation with the Monte-Carlo method

We estimated the uncertainty of the fraction of electron charge transferred to the molecule $\delta$ and from the molecule $\Delta$, as well as the calculated charge transfer times using the standard Monte-Carlo (MC) sampling method. For example, to obtain the estimate of the probability distribution of the fraction of electron charge transferred to the molecule $\delta$, we randomly sampled the energies

on the right hand side of Eq. (9), i.e. input parameters, with N = $10^5$ samples, assuming a normal distribution of each of the terms. Similarly, we have performed MC sampling to obtain the estimate of the probability distribution of the fraction of electron charge transferred from the molecule $\mathit{\Delta}$ using Eq. (14). Charge transfer times were sampled using the equation $\tau = \frac{1-\delta}{\delta}\tau_{CH}$, as discussed in the main text. The core-hole lifetime $\tau_{CH} = (5.4\ \pm 0.4)\ fs$, as deduced from the reported N 1s linewidth of (123 ± 10) meV,[5–7] was used in the calculation. In all cases, the estimate of the output parameter was determined from the cumulative probability distribution at the 50th percentile (median), whereas the lower (upper) bound of the uncertainty was determined at the 16th (84th) percentile.

**Table S2.** Measured Auger kinetic energies used for the calculation of the fraction of electron charge transferred to the molecule $\delta$ and from the molecule $\mathit{\Delta}$ and the corresponding charge transfer times $\tau$.

| **Quantity** | **Value** | **Comment** |
|---|---|---|
| ${}^{multi}E_k(free)$ | (374.75 ± 0.05) eV | this work, from Figure S5 |
| ${}^{multi}E_k(LUMO)$ | (377.68 ± 0.05) eV | this work, from Figure S5 |
| ${}^{ML}E_k(free)$ | (376.94 ± 0.05) eV | this work, from Figure S5 |
| ${}^{ML}E_k(LUMO)$ | (377.76 ± 0.05) eV | this work, from Figure S5 |
| $\Delta E_{screen}(1h)$ | (0.43 ± 0.05) eV | this work, from Figure S3 |
| $\delta$ | 0.43 ± 0.06 | Fraction of electron charge injected to the molecule; from Eq. (9) |

| | | |
|---|---|---|
| $\Delta$ | $0.17 \pm 0.05$ | Fraction of electron charge transferred from the molecule; from Eq. (14) |
| $\tau_{CH}$ | $(5.4 \pm 0.4)$ fs | N 1s core-hole lifetime, from ref. [5-7] |
| $\tau(\mathrm{Au} \rightarrow \pi^*(\pi)_{\boldsymbol{CN}})$ | $7.0^{+2.2}_{-1.5}$ fs | $\tau = \frac{1-\delta}{\delta}\tau_{CH}$, see main text |
| $\tau(\pi^*(\pi)_{\boldsymbol{CN}} \rightarrow \mathrm{Au})$ | $27^{+11}_{-8}$ fs | $\tau = \frac{1-\Delta}{\Delta}\tau_{CH}$, see main text |

## STM and LEED

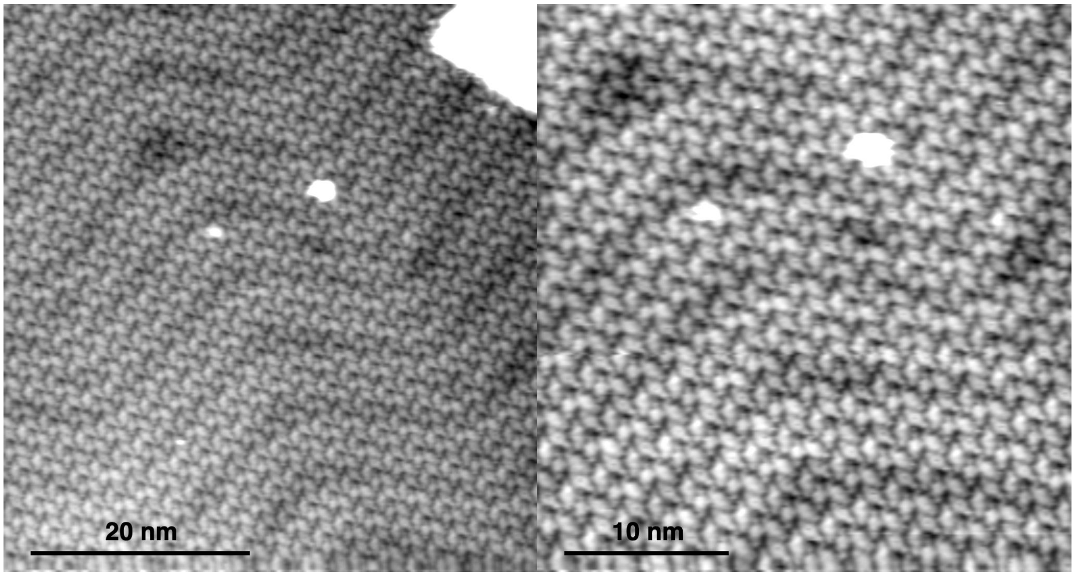


**Figure S7.** STM topographic image of PDIF-$CN_2$ monolayer on Au(111) taken at -1.75 V bias voltage and 230 pA tunneling current.

The molecular domains shown in Figure S7 display a nearly rectangular superlattice, where the short edge is aligned to the [1-10] substrate direction, with an average length of 2.1 ± 0.1 nm. The long edge is misoriented by 5°-6° from the substrate [1-12] direction, with a length of 3.0 ± 0.1

nm. Low energy electron diffraction measurements, Figure S8, reveal a complex superlattice pattern, where one set of fractional order peaks lies along the [1-10] direction, while a symmetric peak splitting by ± 5°-6° is observed along the [1-12] direction, in good agreement with the misalignment of the long edge superlattice observed by STM. Overall, the superlattice appears to be incommensurate with the substrate. This is an evidence of very small substrate interaction, as confirmed in STM images by persistence of the Au(111) herringbone reconstruction, which modulated the topographic contrast.

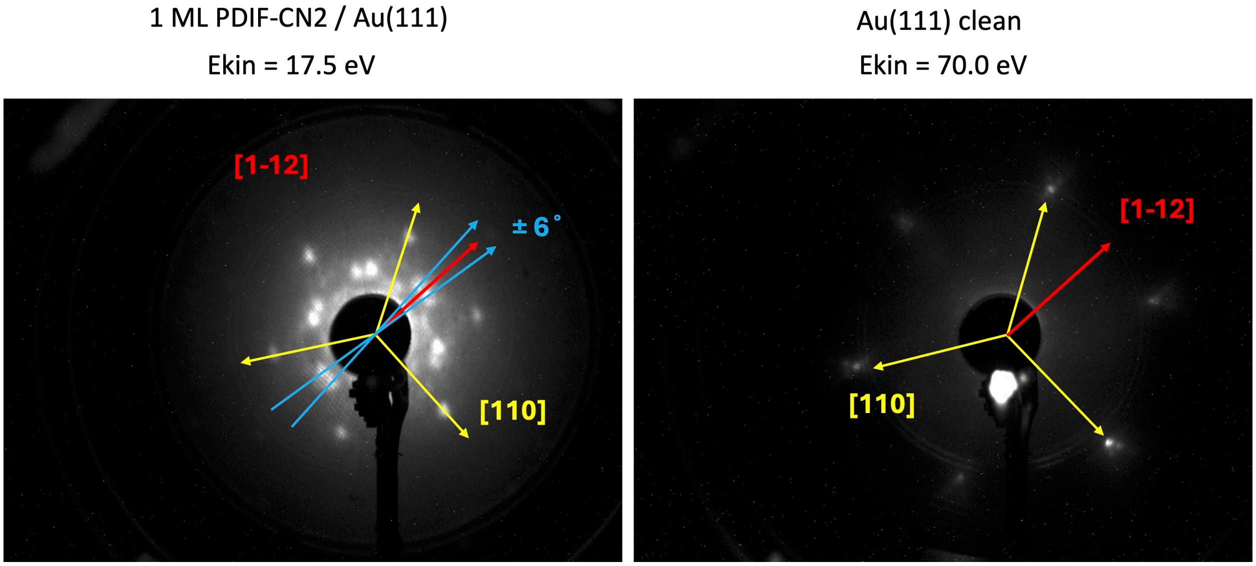


**Figure S8.** a) LEED image of PDIF-$CN_2$ monolayer on Au(111). b) LEED pattern of clean Au(111).

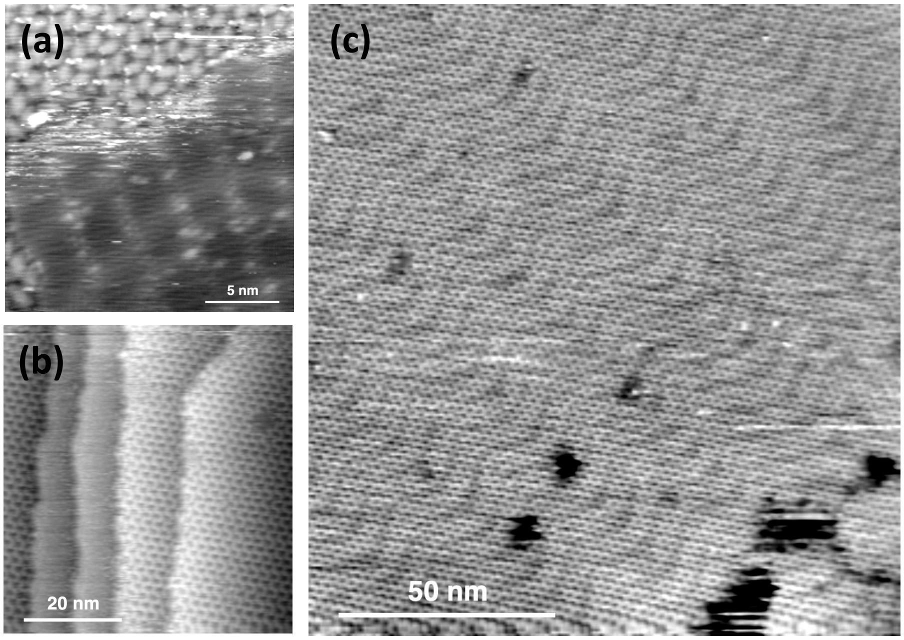


**Figure S9.** STM images of PDIF-$CN_2$ films on Au(111) at increasing molecular coverage. **(a)** At low submonolayer coverage, PDIF-$CN_2$ molecules nucleate into small islands already exhibiting the same phase symmetry as the saturated monolayer. The surrounding bare Au(111) regions display a streaky contrast at island boundaries due to rapidly diffusing molecules on the uncovered surface. In these areas, the characteristic herringbone reconstruction of Au(111), arising from surface stress–induced atomic rearrangement, is clearly resolved. Image taken at +1.6 V bias voltage and 170 pA tunneling current. **(b)** At intermediate monolayer coverage, terraces fully covered by the molecular layer alternate with adjacent terraces that remain devoid of molecular islands, indicating anisotropic growth across step edges. Image taken at +1.9 V bias voltage and 220 pA tunneling current. **(c)** At high monolayer coverage, a continuous molecular film is formed, within which the underlying Au(111) herringbone reconstruction remains visible through the overlayer. Occasional molecular vacancies appear as small dark depressions ("holes") within the

otherwise homogeneous domain, preserving the overall film symmetry. Image taken at +3.1 V bias voltage and 160 pA tunneling current.

## References


(1) Floreano, L.; Naletto, G.; Cvetko, D.; Gotter, R.; Malvezzi, M.; Marassi, L.; Morgante, A.; Santaniello, A.; Verdini, A.; Tommasini, F.; Tondello, G. Performance of the Grating-Crystal Monochromator of the ALOISA Beamline at the Elettra Synchrotron. *Rev. Sci. Instrum.* **1999**, *70* (10), 3855–3864. https://doi.org/10.1063/1.1150001.

(2) Cumpson, P. J.; Seah, M. P. Elastic Scattering Corrections in AES and XPS. II. Estimating Attenuation Lengths and Conditions Required for Their Valid Use in Overlayer/Substrate Experiments. *Surf. Interface Anal.* **1997**, *25* (6), 430–446. https://doi.org/10.1002/(SICI)1096-9918(199706)25:6<430::AID-SIA254>3.0.CO;2-7.

(3) Cossaro, A.; Floreano, L.; Verdini, A.; Casalis, L.; Morgante, A. Comment on "Local Methylthiolate Adsorption Geometry on Au(111) from Photoemission Core-Level Shifts." *Phys. Rev. Lett.* **2009**, *103* (11), 119601. https://doi.org/10.1103/PhysRevLett.103.119601.

(4) Floreano, L.; Cossaro, A.; Gotter, R.; Verdini, A.; Bavdek, G.; Evangelista, F.; Ruocco, A.; Morgante, A.; Cvetko, D. Periodic Arrays of Cu-Phthalocyanine Chains on Au(110). *J. Phys. Chem. C* **2008**, *112* (29), 10794–10802. https://doi.org/10.1021/jp711140e.

(5) Shaw, D. A.; King, G. C.; Read, F. H.; Cvejanovic, D. The Observation of Electric-Dipole-Forbidden Inner-Shell Transitions in N 2 and Ar by the Electron Energy-Loss Technique. *J. Phys. B At. Mol. Phys.* **1982**, *15* (11), 1785–1793. https://doi.org/10.1088/0022-

3700/15/11/023.

(6) Coville, M.; Thomas, T. D. Molecular Effects on Inner-Shell Lifetimes: Possible Test of the One-Center Model of Auger Decay. *Phys. Rev. A* **1991**, *43* (11), 6053–6056. https://doi.org/10.1103/PhysRevA.43.6053.

(7) Ilakovac, V.; Carniato, S.; Gallet, J.-J.; Kukk, E.; Horvatić, D.; Ilakovac, A. Vibrations of Acrylonitrile in N 1s Excited States. *Phys. Rev. A* **2008**, *77* (1), 012516. https://doi.org/10.1103/PhysRevA.77.012516.
